# Research on the Dynamic Mechanism and Multi-Parameter Collaborative Optimization of a Cantilevered Conveyor Trough in Combine Harvesters for Vibration Suppression

**DOI:** 10.3390/s25237397

**Published:** 2025-12-04

**Authors:** Qi He, Zhan Su, Pengfei Qian, Zhong Tang, Zhaoming Zhang, Jiahao Shen, Ting Lu

**Affiliations:** 1 School of Mechanical Engineering, Jiangsu University, Zhenjiang 212013, China; 2 Key Laboratory of Crop Harvesting Equipment Technology of Zhejiang Province, Jinhua University of Vocational Technology, Jinhua 321017, China; 3 School of Agricultural Engineering, Jiangsu University, Zhenjiang 212013, China

**Keywords:** cantilevered conveyor trough, structural parameter optimization, orthogonal experiment, multi-factor analysis of variance, sway characteristics

## Abstract

Excessive swing of the cantilevered conveyor trough is a key issue restricting the working efficiency and operational stability of combine harvesters. To suppress its swing, this study established a dynamic model of the conveyor trough to reveal the influence mechanisms of the initial angle, overall length, and cylinder pivot length on its swing characteristics. Orthogonal experimental design and multi-factor analysis of variance were employed to systematically analyze the significance of these three factors on swing amplitude, identifying cylinder pivot length as the most dominant factor. Optimization results determined the optimal parameter combination as an initial angle of 48.33°, an overall length of 1.45 m, and a cylinder pivot length of 1.1 m. Field tests verified that this optimized scheme reduces the swing amplitude by 11.62%, with a minimal error of 0.57% between theoretical and measured values, providing a reliable theoretical and experimental basis for the low-vibration design of combine harvester conveying mechanisms.

## 1. Introduction

Rice combine harvesters represent pivotal machinery in modern agricultural production, where operational stability and efficiency directly determine grain harvest quality and yield [1,2,3,4]. As the critical transfer component linking the header and threshing unit, the cantilevered conveyor trough plays a decisive role in overall machine stability [5,6,7]. However, complex and variable field operating conditions subject the header to impact loads that propagate trough the cantilevered conveyor trough, inducing severe sway [8,9,10,11]. This phenomenon not only accelerates structural fatigue and compromises operator comfort but also risks material blockage and increases grain entrainment loss [12,13]. Consequently, in-depth investigation of the dynamic characteristics of the cantilevered conveyor trough, coupled with structural parameter optimization to suppress detrimental vibrations, holds significant theoretical and engineering value for enhancing combine harvester performance [14,15,16,17].

Scholars worldwide have explored vibration issues in conveying systems from multiple perspectives [18,19,20,21]. Regarding mechanism analysis and testing, Cieplok’s [22] spatial modeling of vibratory conveyors demonstrated that misalignment between the resultant excitation force vector and the machine’s center of mass causes deviations from ideal trough trajectories, providing theoretical insights into vibration complexity. Hrabovský et al. [23] empirically identified the highest effective vibration velocity in the vertical direction trough sensor measurements, offering critical guidance for sensor placement and data interpretation. Jing et al.’s [24] research directly addressed noise generation in combine harvester conveyor troughs, proposing damping optimization strategies that validated dynamic behavior modification as an effective solution pathway. Nevertheless, these studies predominantly focused on theoretical models or specific phenomena, failing to elucidate the intrinsic correlations and action mechanisms between structural parameters of the cantilevered conveyor trough and its holistic sway characteristics [25,26,27,28].

In structural optimization, multi-parameter collaborative design has emerged as a core methodology for complex engineering problems [29,30,31]. In agricultural machinery, Yu et al. [32] optimized cutting components of garlic harvesters, while Huang et al. [33] enhanced Boehmeria nivea harvester cutting–conveying mechanisms trough multi-factor regression models, with both demonstrating substantial performance gains. For structurally analogous components like crane telescopic booms [34,35,36] and excavator booms [37,38], researchers have successfully applied multi-body dynamics and reliability-based optimization to achieve weight reduction and dynamic stability improvements [39]. These methodologies find parallel applications in other sectors involving heavy machinery. Likewise, equipment such as high-capacity belt conveyors, bucket-wheel excavators, and scraper conveyors encounter comparable challenges of dynamic instability and structural vibration [40,41,42]. Therefore, the methodological advances made in these fields offer a valuable foundation for present research.

Despite this progress, critical gaps persist in optimizing the cantilevered conveyor trough of combine harvesters [43,44,45]. Existing research predominantly targets static strength, fatigue life, or noise control, such as Wei et al.’s [46] static force analysis of swing arms, while neglecting dynamics-focused optimization explicitly targeting sway suppression. Although multi-parameter optimization precedents exist for analogous components, the systematic application of statistical methods to identify and optimize key parameters of the cantilevered conveyor trough remains unexplored [47,48,49,50]. This methodological gap impedes precise quantification of each parameter’s contribution ratio to sway amplitude, thereby hindering targeted collaborative optimization [51,52,53].

To address these limitations, this study conducts a systematic investigation of vibration suppression in the cantilevered conveyor trough of rice combine harvesters, spanning theoretical modeling, parameter identification, and experimental validation. A dynamic model is established to dissect the physical origins of sway. Orthogonal experimental design is employed with initial angle, overall length, and cylinder pivot length as critical optimization parameters, which reduces experimental iterations while ensuring statistical rigor. Multi-factor analysis of variance (ANOVA) quantifies each parameter’s contribution ratio to sway amplitude to identify dominant factors. Field validation tests verify the efficacy of the optimized parameter combination, providing theoretical foundations and engineering solutions for low-vibration design and multi-parameter collaborative optimization of cantilevered conveyor troughs in combine harvesters.

## 2. Materials and Methods

### 2.1. Output Response Test of the Cantilevered Conveyor Trough

#### 2.1.1. Experimental Device and Instruments

**Figure 1:**
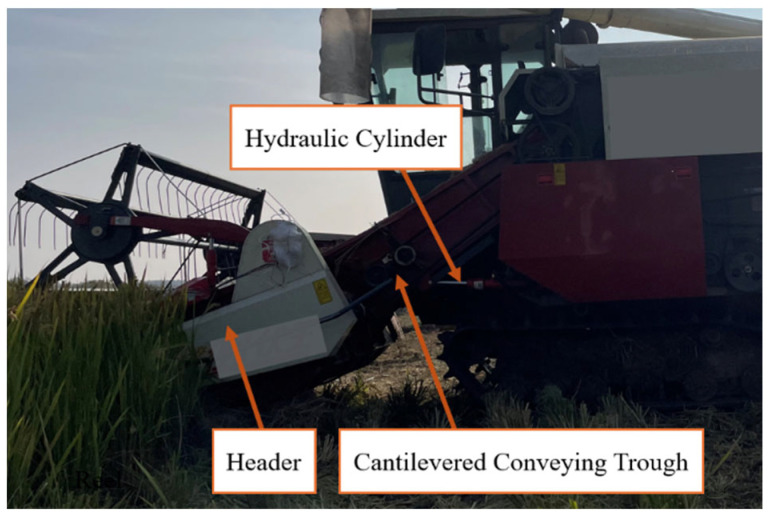
Cantilevered conveyor trough entity.

**Figure 2:**
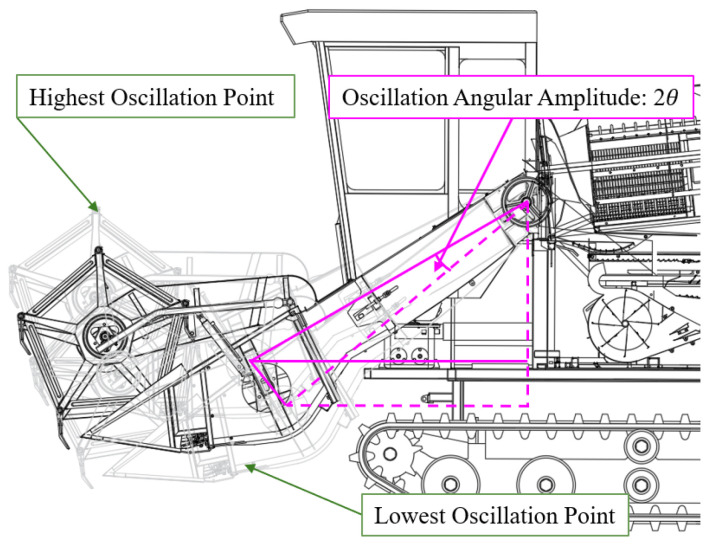
Mathematical simplified model of cantilevered conveyor trough.

This study focuses on the cantilevered conveyor trough, a critical component of combine harvesters responsible for conveying rice stalks from the header to the threshing drum. Its structural assembly comprises the trough body, hydraulic cylinder support system, and chassis connection elements. Physical configuration is illustrated in Figure 1, while Figure 2 presents a mathematically simplified model derived from dynamic equations for analyzing the influence of parameters such as overall length and initial angle on sway behavior.

**Figure 3:**
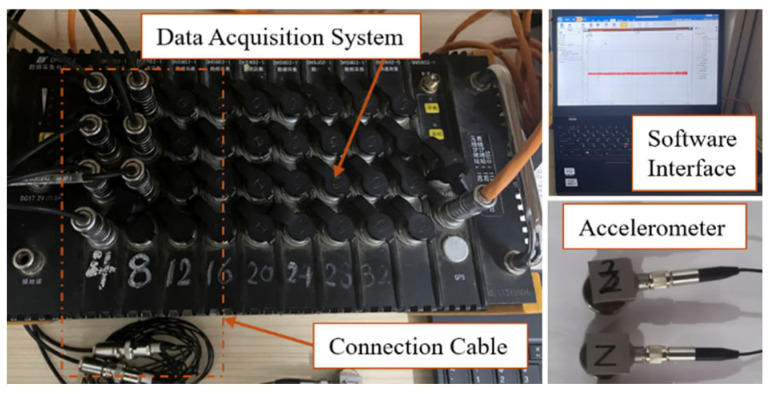
Dynamic signal acquisition analyzer.

Vibration data acquisition employed a system developed by Donghua Testing Technology Co., Ltd. (Jingjiang, China), featuring a DH5902 dynamic signal analyzer, dedicated software, and triaxial accelerometers, as shown in Figure 3. The complete setup included one computer, one analyzer unit, and connecting cables. The analyzer supports 38 signal channels (36 vibration channels and 2 rotational speed channels) with IEPE input mode. A sample frequency of 1 kHz was configured to ensure comprehensive capture of high-frequency vibration data. Triaxial accelerometers (Model 1A312E) with a measurement range of 500 m/s^2^, resolution of 0.001 g, and sensitivity of 1 mV/m/s^2^ were utilized. Equipment parameter adjustments included channel configuration, filter settings, and sensitivity calibration.

#### 2.1.2. Layout of Sensor Test Points

**Figure 4:**
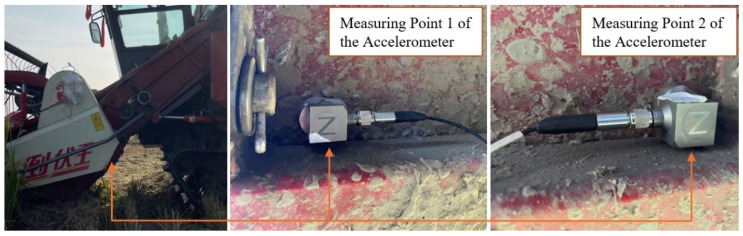
Verification test instrument layout.

This experiment prioritized analyzing dynamic responses of the cantilevered conveyor trough under varying structural parameters. Field tests were conducted in agricultural fields of Dantu District, Zhenjiang City, Jiangsu Province. The combine harvester was operated at constant speed with full cutter bar width engagement. Two symmetric test points were positioned at the junction between the trough and chassis to minimize random errors, as this symmetric layout helps average out asymmetries in vibration transmission caused by uneven loads or structural tolerances, ensuring that the acquired acceleration signals accurately represent the overall sway characteristics of the cantilevered conveyor trough, as shown in Figure 4.

### 2.2. Theoretical Foundation and Model Construction for Structural Optimization Theoretical Foundation and Model Construction for Structural Optimization

#### 2.2.1. Theoretical Model Development

Dynamic analysis of Equation (1) reveals that the overall length and initial angle of the conveyor trough are pivotal factors inducing sway [54]. To reduce sway amplitude during operation and prevent crop blockage, structural redesign of the trough is essential. The initial angle directly governs the inclination angle at the junction between the trough and header, thereby influencing rice stalk conveyance efficiency. An excessively small initial angle increases blockage risk due to excessive junction inclination, while an overly large angle extends the trough length, compromising machine maneuverability. Consequently, theoretical modeling must optimize the overall length, cylinder pivot length, and structural inclination angle in accordance with the physical constraints of the header–conveyor interface.

(1) $$T=\frac{7}{24}ml^{2}{\dot{\theta_{1}}}^{2}$$

where *T* is the total kinetic energy of the system, *m* is the conveyor chute mass, *l* is the overall length, and *θ*_1_ is the initial angle.

**Figure 5:**
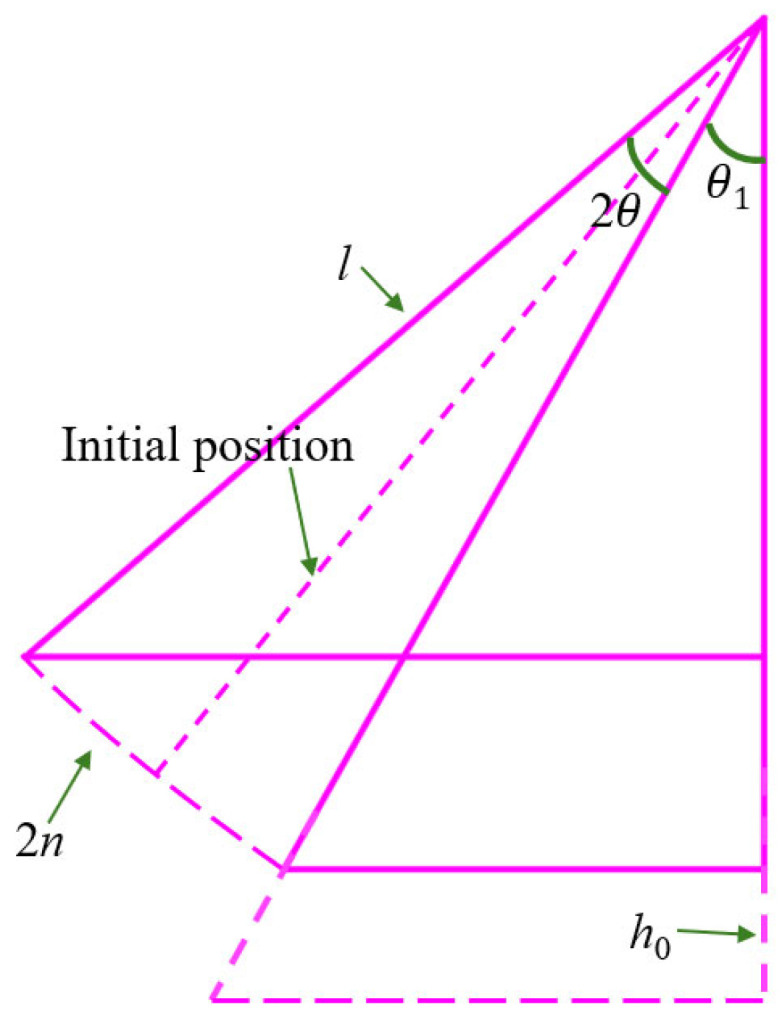
Simple model of cantilevered conveyor trough.

To address sway imbalance caused by length and initial angle variations, the parameter set for the simplified dynamics model of the header–conveyor system is completed as illustrated in Figure 5. This model assumes that during combine harvester operation, the header ground clearance (*h*_0_) is adjusted via hydraulic cylinders to achieve an initial angle (*θ*_1_). Under this configuration, the trough swing angle range is 2*θ*, with a corresponding arc length of 2*n*. This sway amplitude range serves as a constraint condition for post-optimization trough design.

**Figure 6:**
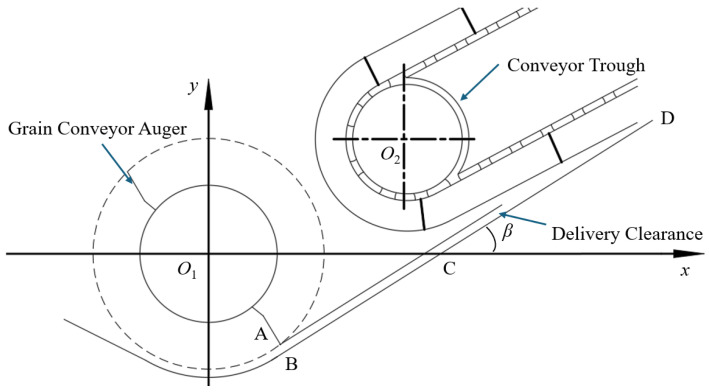
Relative position of the auger and the conveyor trough.

Ensuring seamless material transfer from header to trough requires optimizing the geometric relationship at their junction to minimize collisions between rice stalks and scraper plates. As shown in Figure 6, a parametric model aligns the grain auger base plate plane (BC) with the conveyor trough base plate (CD) on a common plane, guaranteeing continuous stalk transfer. In this model, Equation (2) for line BCD is defined as follows:

(2) y=tanβ·x+e

where *β* denotes the structural inclination angle between the trough base plate and the *x*-axis, and *e* represents the *y*-axis intercept.

#### 2.2.2. Limit Inclination Angle Calculation and Parameter Validation

Determining the optimal structural inclination angle requires satisfying distance constraints from the auger center (*O*_1_) and trough driven shaft center (*O*_2_) to line BCD. The geometric relationship is governed by Equation (3):

(3) tanβ·xO1−yO1+etan2β+1=R1+lABtanβ·xO2−yO2+etan2β+1=R2+ljxtanβ>0e<0

where *O*_1_ coordinates are (0, 0), *O*_2_ coordinates are (30, 20) (unit: mm), *l_AB_* is the minimum distance from auger paddle tip to base plate (20 mm), and *l_gx_* is the conveyance clearance (15 mm). Solving yields a practical *β* value of 41.67° and *θ*_1_ of 48.33°, in close agreement with the actual initial angle (46.40°) of the combine harvester. This configuration maintains adequate trough rotational speed for stalk conveyance and ensures smooth transition between header and trough, where *e* is −26.8, yielding Equation (4):

(4) $$y=\frac{24+\sqrt{51}}{35}x-26.8$$

This line defines the limiting installation position for the trough base plate. To mitigate experimental variability, three initial angle levels (46.40°, 48.33°, 50°) are selected for multi-parameter optimization validation.

**Figure 7:**
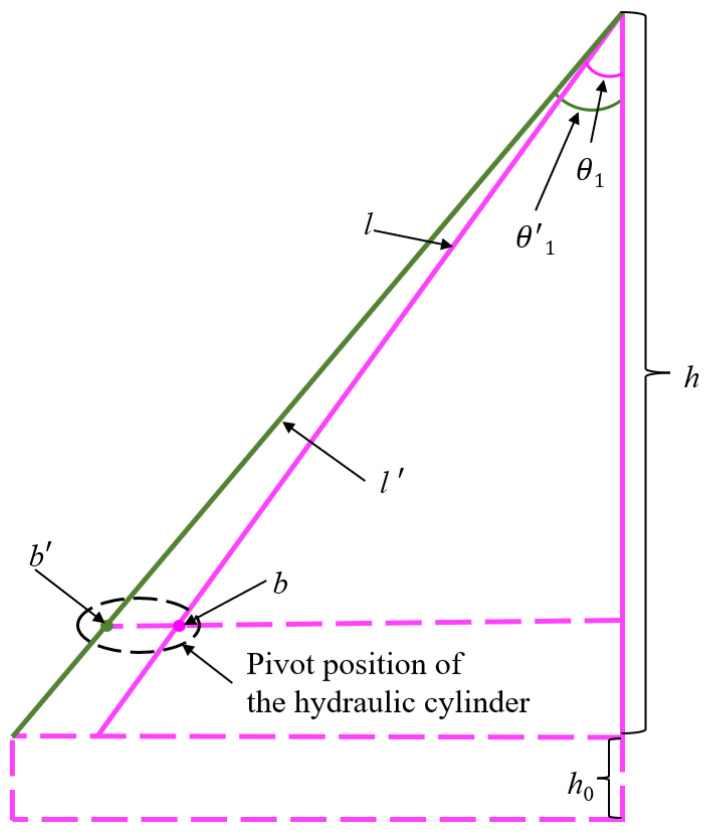
Comparison of the cantilevered conveyor trough model before and after optimization.

Fulfilling the smooth transition requirement further determines the overall length and cylinder pivot length. Optimized and pre-optimization models of the cantilevered conveyor trough are constructed as depicted in Figure 7. Applying a geometric constraint gives Equation (5):

(5) $$\left\{\begin{array}{c} \cos\theta_{1}\prime =\frac{h}{l\prime }\\ \frac{b}{l}=\frac{b\prime }{l\prime } \end{array}\right.$$

where *l*′ is the optimized overall length, *b* is the cylinder pivot length, *b*′ is the optimized cylinder pivot length, and *θ*′ is the optimized initial angle. With *h* set to 1 m, the calculations yield *l*′ at 1.50 m and *b*′ at 1.04 m.

### 2.3. Orthogonal Experimental Design and Multi-Factor ANOVA Methodology

To systematically analyze the influence of cantilevered conveyor trough structural parameters on sway amplitude, this study employs a multi-factor experimental design. Based on preceding analysis, initial angle (Factor A), overall length (Factor B), and cylinder pivot length (Factor C) were identified as critical factors. Each factor was assigned three levels as shown in Table 1, balancing structural feasibility and experimental practicality: initial angle at 46.40°, 48.33°, and 50°; overall length at 1.45 m, 1.504 m, and 1.55 m; cylinder pivot length at 1.00 m, 1.04 m, and 1.10 m. This range encompasses operational parameter variations observed in field conditions.

**Figure 8:**
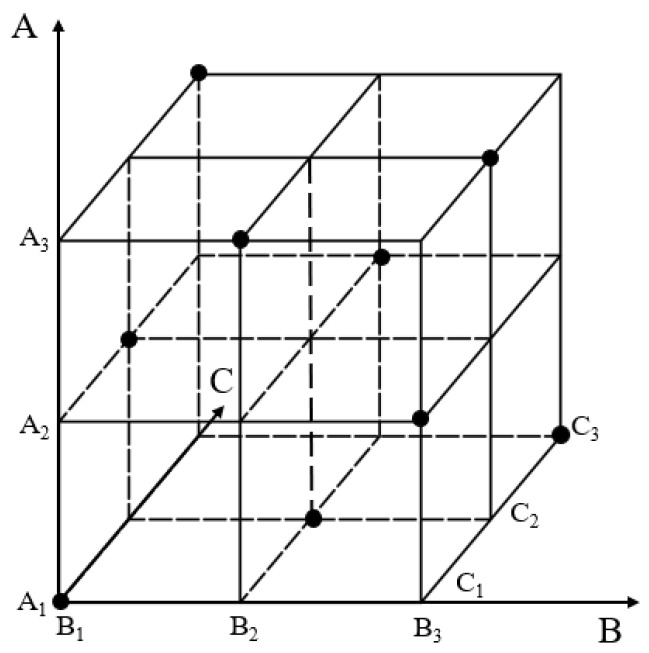
Orthogonal experimental model.

A full factorial design would require 27 combinations. To balance experimental efficiency with comprehensiveness, an orthogonal experimental design was adopted to reduce test iterations. This approach samples representative points across the parameter space design model in Figure 8, selecting nine critical experiments from the full set. This ensures uniform distribution of factor levels, enabling efficient evaluation of main effects and potential interactions, as shown in the experimental scheme in Table 2.

Experimental data were obtained through numerical simulation. Each parameter combination was substituted into the cantilevered conveyor trough dynamic differential Equation (6), which was solved in MATLAB2022b to determine steady state swing angle variations. The sway amplitude at the trough end was calculated by multiplying the swing angle variation by the overall length. Given the overall length substantially exceeds swing angle variations, the amplitude was approximated as the sway amplitude at the trough end using the geometric relationship in Equation (7):

(6) $$\begin{array}{c} \frac{7}{12}ml^{2}\ddot{\theta }+\frac{1}{2}kb^{2}\left[2\sin\left(\theta +\theta_{1}\right)\cos\left(\theta +\theta_{1}\right)-2\cos\left(\theta +\theta_{1}\right)\sin\theta_{1}\right]\\ +\frac{1}{2}mgl\sin\left(\theta +\theta_{1}\right)=Fl-cb^{2}\dot{\theta }{\left[\cos\left(\theta +\theta_{1}\right)\right]}^{2} \end{array}$$

(7) S=θ·l

where *S* denotes sway amplitude (unit: m), *θ* represents swing angle variation (unit: rad), and *l* is the overall length (unit: m). Swing angle response curves were generated via simulation for each parameter combination, with sway amplitude results aggregated for subsequent analysis. To minimize random errors, each experiment was repeated three times, and the mean sway amplitude served as the final observed value. Data acquisition focused exclusively on displacement responses at the trough end, with high-frequency noise interference excluded from analysis.

Statistical analysis implemented multi-factor ANOVA using IBM SPSS Statistics 22.0 software. Sway amplitude served as the dependent variable, with initial angle, overall length, and cylinder pivot length as fixed factors. A main effects model was configured to initially evaluate independent factor influences, excluding interaction terms. The significance threshold *α* was set at 0.05. Prior to analysis, homogeneity of variance was verified using Levene’s test to satisfy ANOVA assumptions. Duncan’s multiple range test compared subset differences across factor levels and validated orthogonality principles. Model goodness-of-fit was assessed via the adjusted coefficient of determination (R^2^ = 0.990), while residual analysis confirmed model reliability. This quantified each parameter’s contribution ratio to sway amplitude, providing statistical foundations for optimization design.

## 3. Results and Discussion

### 3.1. Multi-Factor ANOVA Results and Optimization Validation for the Cantilevered Conveyor Trough Structure

#### 3.1.1. Influence Degree Analysis Under Multi-Parameter Combinations

**Figure 9:**
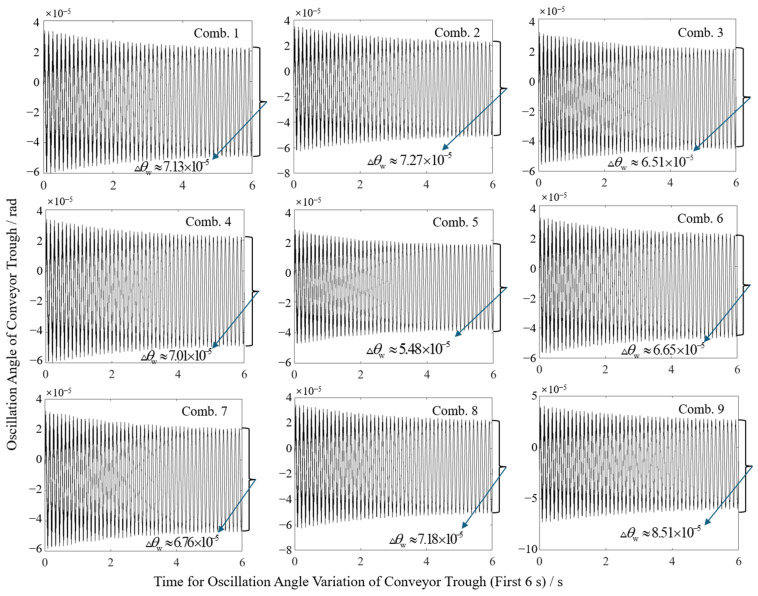
Schematic diagram of change in lower swing angle in different horizontal combinations.

Orthogonal experimental results reveal the significant impacts of structural parameters on sway characteristics, as shown in Figure 9. Compiled swing angle variations and end sway amplitudes for all test groups are presented in Table 3. Simulation results demonstrate sway amplitudes ranging from 0.079 mm to 0.132 mm. The minimum amplitude occurred at combination A_2_B_2_C_3_ (initial angle 46.40°, overall length 1.45 m, cylinder pivot length 1.10 m), while the maximum occurred at A_3_B_3_C_2_ (initial angle 50°, overall length 1.55 m, cylinder pivot length 1.00 m). This disparity preliminarily confirms the critical role of parameter optimization in sway suppression.

Parameter combinations and corresponding sway amplitudes were imported into SPSS. Between-subject factors are detailed in Table 4. For initial angle, each level comprises three cases, yielding a total of nine experiments. Identical case distributions were applied to overall length and cylinder pivot length, validating the orthogonality and comprehensiveness of the experimental design. Between-subject effect testing, as shown in Table 5, was used to evaluate whether mean values across subgroups exhibited statistically significant differences under varying control variable levels, with sway amplitude as the dependent variable.

ANOVA-quantified factors influence magnitudes. Significance levels for initial angle, overall length, and cylinder pivot length were 0.032, 0.036, and 0.023 (all < 0.05), confirming statistically significant effects on sway amplitude. Comparing Type III sums of squares and F-values, cylinder pivot length exerted the strongest influence (F = 42.250), followed by initial angle (F = 30.484) and overall length (F = 27.062). This aligns with dynamic model predictions: hydraulic cylinder pivot position directly alters torque distribution, dominating sway behavior. To ensure smooth transition between trough and header and prevent rice stalk blockage, the initial angle was fixed at 48.33°.

#### 3.1.2. Interaction Analysis Under Multi-Parameter Combinations

Duncan’s post hoc test results for the three factors are presented in Table 6. Each parameter level was assigned to distinct subsets with no overlap between subsets, confirming significant inter-level differences. Notably, an initial angle of 48.33° (sway amplitude 0.104 mm) satisfies smooth transition design requirements, minimizing blockage risk without substantially increasing sway amplitude and demonstrating multi-objective optimization feasibility.

**Figure 10:**
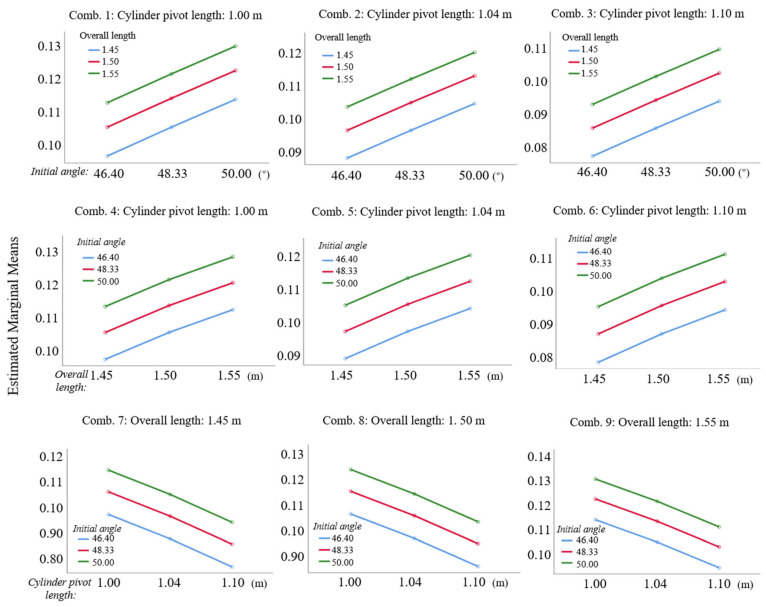
Relationship between different main factors and estimated marginal average.

For interaction analysis, we employed estimated marginal means, as shown in Figure 10. Non-intersecting and non-converging lines for all factors indicate no significant interactions between initial angle, overall length, and cylinder pivot length. Sway amplitude trends remained consistent across overall length levels with negligible slope variations. This supports the main effects of model independence, enabling single-factor optimization and streamlining of parameter design.

#### 3.1.3. Optimization Validation Under Fixed Initial Angle

Mean sway amplitudes were 0.096 mm, 0.105 mm, and 0.112 mm for overall length levels of 1.45 m, 1.50 m, and 1.55 m, respectively, preliminarily identifying 1.45 m as optimal. For cylinder pivot length levels of 1.00 m, 1.04 m, and 1.10 m, mean amplitudes were 0.114 mm, 0.105 mm, and 0.094 mm, preliminarily identifying 1.10 m as optimal. To verify robustness against transient motion effects, full factorial experiments were conducted with the initial angle fixed at 48.33°, as shown in Table 7.

**Figure 11:**
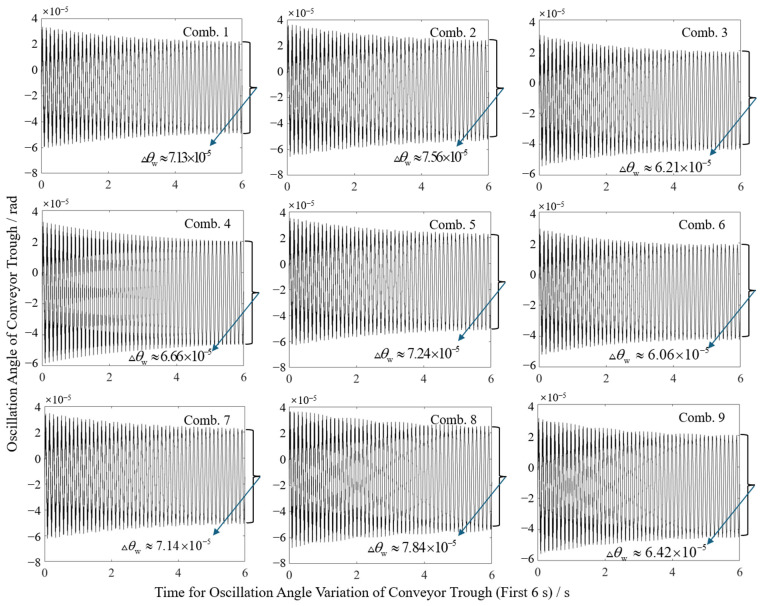
Variation in swing angle in different horizontal combinations.

**Figure 12:**
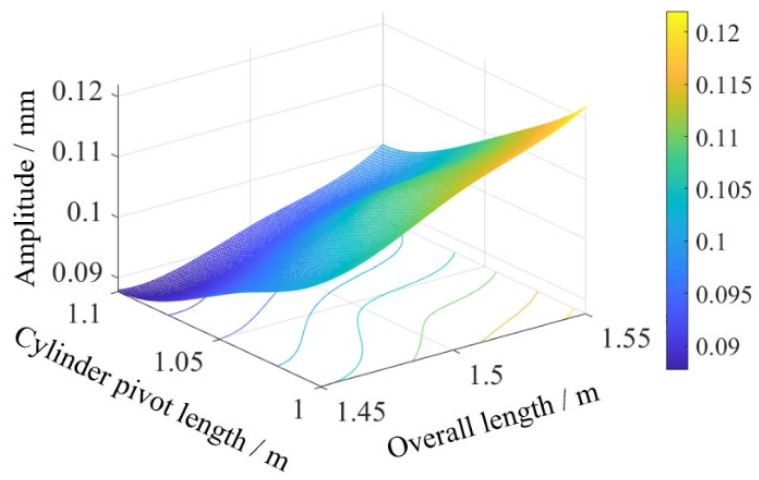
Contour map of combined swing.

Swing angle results from Figure 11 and statistical summaries from Table 8 show that combination 6 (overall length 1.45 m, cylinder pivot length 1.10 m) achieved a minimum sway amplitude of 0.088 mm, while combination 8 (overall length 1.55 m, cylinder pivot length 1.00 m) yielded a maximum of 0.122 mm. Contour plot visualization in Figure 12 reveals sway amplitude gradients: amplitude decreases with reduced overall length or increased cylinder pivot length, with the latter exhibiting higher sensitivity. This higher sensitivity mechanically stems from the extended moment arm of the hydraulic force when the cylinder pivot length is increased, which optimizes torque distribution and damping in the dynamic system, as described in Equation (6). Compared to the theoretical sway amplitude of 0.0979 mm for the original configuration (initial angle 46.40°, overall length 1.45 m, cylinder pivot length 1.00 m), the optimized combination (initial angle 48.33°, overall length 1.45 m, cylinder pivot length 1.10 m) reduced the amplitude to 0.088 mm, representing a 10.11% decrease.

This validates optimization efficacy and underscores cylinder pivot length’s pivotal role in sway control. The reduction mechanism likely involves modified hydraulic cylinder force arm ratios that optimize torque equilibrium, thereby suppressing vibration amplitude. The initial angle of 48.33° achieves equilibrium between smooth material transition and sway control, exemplifying engineering compromise under multi-constraint optimization.

### 3.2. Field Validation and Dynamic Characteristic Analysis

**Figure 13:**
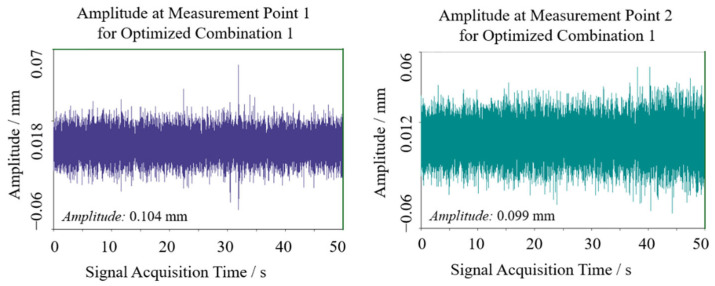
Swing results of optimization 1 test point.

**Figure 14:**
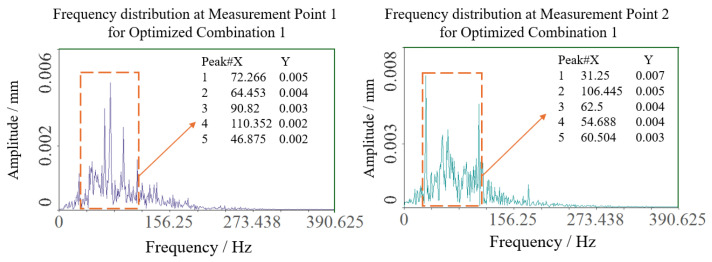
Spectrum results of optimization 1 test point.

Field experiments were conducted to verify the practical efficacy of theoretically optimized parameters. The cantilevered conveyor trough initial angle was adjusted to 48.33° (optimization combination 1) while maintaining other parameters constant. Two symmetric sensor test points were positioned at the junction between trough and chassis to ensure data reliability. Signals were acquired during stable operational conditions, with transient segments at signal onset and termination excluded. Stable working interval data underwent double integration and FFT transformation to obtain sway amplitude and vibration frequency distribution, as shown in Figure 13 and Figure 14. Test point 1 exhibited an amplitude of approximately 0.104 mm, while test point 2 showed 0.099 mm, yielding a mean amplitude of 0.1015 mm. Vibration frequencies were concentrated in the 50–110 Hz range, falling within the low-frequency regime relative to the structural characteristics of harvesters, with dominant frequencies of 72.226 Hz at test point 1 and 31.25 Hz at test point 2. The differences in vibration spectra between the test points may be attributed to field test variability, such as asymmetric loading from uneven crop distribution, minor structural tolerances, or environmental factors like ground irregularities. However, consistency in mean amplitude values across points supports the robustness of the optimization outcomes, as the low-frequency sway characteristics remain dominant and aligned with the theoretical model.

**Figure 15:**
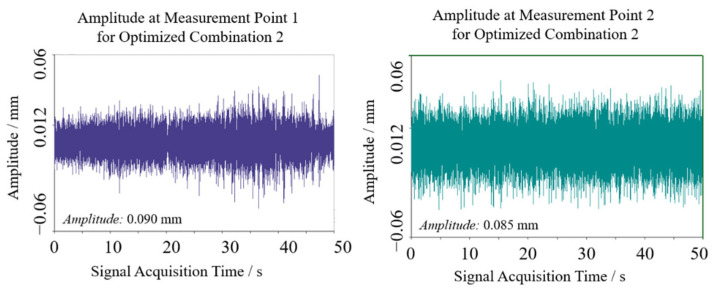
Swing results of optimization 2 test point.

**Figure 16:**
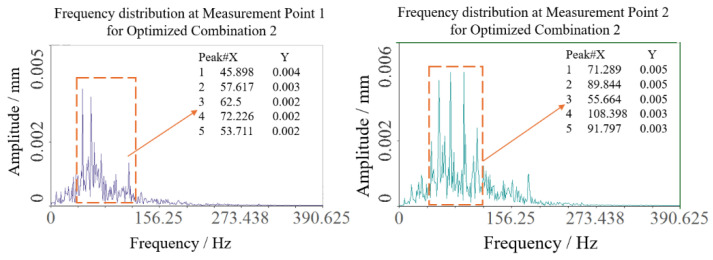
Spectrum results of optimization 2 test point.

Similarly, optimization combination 2 yielded sway amplitudes of 0.090 mm and 0.085 mm at the two test points, with a mean of 0.0875 mm, as shown in Figure 15 and Figure 16. Vibration frequencies remained predominantly low-frequency, ranging from 50 to 110 Hz, with dominant frequencies of 45.898 Hz at test point 1 and 71.289 Hz at test point 2.

**Figure 17:**
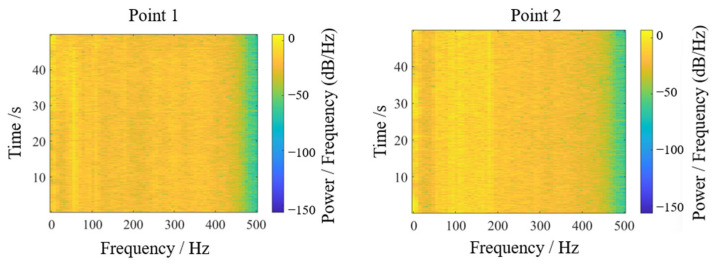
Displacement signal energy diagram.

**Figure 18:**
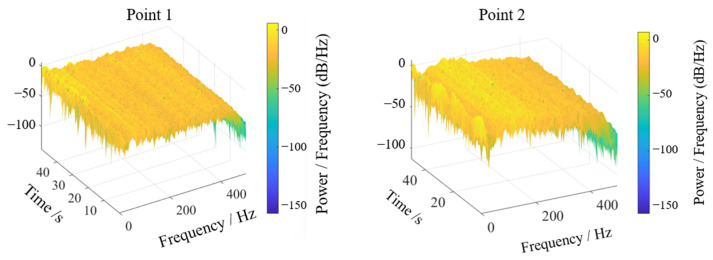
Three-dimensional waterfall diagram of displacement signal energy.

Energy-based vibration analysis was performed via Fourier transformation of acceleration signals, generating an energy response spectrum in the sway direction, as shown in Figure 17 and Figure 18. For test point 1, displacement signal energy concentrated at low frequencies near 50 Hz perpendicular to the trough sway direction. The three-dimensional spectrogram provided intuitive energy distribution visualization: despite minor environmental and experimental interference, persistent dominant frequency peaks were evident over time. Test point 2 similarly showed concentrated low-frequency energy near 70–100 Hz, consistent with prior analysis.

To evaluate the accuracy and error between the original sway amplitude of 0.099 mm, the theoretical design amplitude, and the optimized design amplitude, and to assess the validity of theoretical optimization and the reliability of experiments, the parameter combinations and sway amplitudes under all three conditions were statistically compared using data from Table 9. Under the dual optimization objectives of seamless material conveyance and sway reduction, optimization combination 2 achieved a theoretical sway amplitude of 0.088 mm and an actual amplitude of 0.0875 mm, yielding a 0.57% error. This represents an 11.62% reduction compared to the original parameter combination, which had an amplitude of 0.099 mm. These results validate the effectiveness of multi-parameter collaborative optimization, demonstrating that the strategic configuration of cylinder pivot length and overall length significantly enhances the dynamic performance of the conveyor trough. Minor errors may stem from factors such as sensor measurement noise, slight environmental variations during field tests, or simplifications in the dynamic model; however, the low error rates consistently support the robustness of the approach.

## 4. Conclusions

This study systematically investigated the influence of structural parameters on the sway characteristics of the cantilevered conveyor trough through integrated theoretical modeling, orthogonal experimental design, multi-factor ANOVA, and field validation. The following conclusions were drawn:

1. Cylinder pivot length exerted the most significant influence on sway amplitude (F = 42.250, *p* = 0.023), surpassing the contributions of initial angle (F = 30.484) and overall length (F = 27.062). This confirms that repositioning the hydraulic cylinder pivot directly optimizes torque distribution, effectively suppressing vibration. Duncan’s post hoc test further validated statistically significant differences between factor levels, providing robust statistical foundations for engineering optimization.
2. The optimal parameter combination identified via orthogonal design (initial angle 48.33°, overall length 1.45 m, cylinder pivot length 1.10 m) reduced the theoretical sway amplitude to 0.088 mm, representing a significant 11.62% decrease compared to the original configuration, which had an amplitude of 0.099 mm. Field validation confirmed an actual sway amplitude of 0.0875 mm under this combination, yielding a negligible 0.57% deviation from theoretical predictions. This demonstrates the exceptional robustness and reliability of the optimization framework.
3. Setting the initial angle to 48.33° achieved dual objectives: sway amplitude reduction and seamless geometric transition between header and trough. This configuration minimized rice stalk blockage risks by ensuring continuous alignment between the grain auger base plate and cantilevered conveyor trough base plate. The optimized design exemplifies feasible engineering trade-offs under multi-constraint conditions, validating the practicality of integrated structural optimization for agricultural machinery.
4. The study emphasizes the application of sensor data for structural optimization. The dynamic model incorporated simplifications under ideal assumptions, such as neglecting elastic deformation and nonlinear effects; however, the conclusions verify its effectiveness within the engineering scope. The applicable parameter ranges are initial angle 46.4–50°, overall length 1.45–1.55 m, and cylinder pivot length 1.00–1.10 m, which are suitable for combine harvesters operating at constant speed on relatively flat terrain. Deviations beyond these ranges may require revalidation. Future work will address actual device characteristics and complex field conditions to enhance model generality.

## Figures and Tables

**Table 1:** Horizontal code of orthogonal experimental factors.

Factors/Levels	Initial Angle (A)/°	Overall Length (B)/m	Cylinder Pivot Length (C)/m
1	48.33	1.50	1.04
2	46.40	1.45	1.00
3	50.00	1.55	1.10

**Table 2:** Orthogonal experimental scheme.

Run Number	Level Combination	Initial Angle/°	Overall Length/m	Cylinder Pivot Length/m
1	A_1_B_1_C_1_	48.33	1.50	1.04
2	A_1_B_2_C_2_	48.33	1.45	1.00
3	A_1_B_3_C_3_	48.33	1.55	1.10
4	A_2_B_1_C_2_	46.40	1.50	1.00
5	A_2_B_2_C_3_	46.40	1.45	1.10
6	A_2_B_3_C_1_	46.40	1.55	1.04
7	A_3_B_1_C_3_	50.00	1.50	1.10
8	A_3_B_2_C_1_	50.00	1.45	1.04
9	A_3_B_3_C_2_	50.00	1.55	1.00

**Table 3:** Swing results of each combination under orthogonal experiments.

Level Combination	A_1_B_1_C_1_	A_1_B_2_C_2_	A_1_B_3_C_3_	A_2_B_1_C_2_	A_2_B_2_C_3_	A_2_B_3_C_1_	A_3_B_1_C_3_	A_3_B_2_C_1_	A_3_B_3_C_2_
Swing angle (×10^−5^ rad)	7.13	7.27	6.51	7.01	5.48	6.65	6.76	7.18	8.51
Swing amplitude/mm	0.107	0.105	0.101	0.105	0.079	0.103	0.102	0.104	0.132

**Table 4:** Parameters of inter-subject factors.

	Initial Angle/°	Overall Length/m	Cylinder Pivot Length/m	Swing Amplitude/mm	CARD	Number of Configurations		
1	48.33	1.50	1.04	0.107	1	Initial angle	46.40	3
2	48.33	1.45	1.00	0.105	2		48.33	3
3	48.33	1.55	1.10	0.101	3		50.00	3
4	46.40	1.50	1.00	0.105	4	Overall length	1.45	3
5	46.40	1.45	1.10	0.079	5		1.50	3
6	46.40	1.55	1.04	0.103	6		1.55	3
7	50.00	1.50	1.10	0.102	7	Cylinder pivot length	1.00	3
8	50.00	1.45	1.04	0.104	8		1.04	3
9	50.00	1.55	1.00	0.132	9		1.10	3

**Table 5:** Inter-subject effect test.

Tests of Between-Subject Effects						Grand Mean	
	Type III sum of squares	Degrees of freedom	Mean square	F-value	Significance	Mean	0.104
Modified model	0.001	6	0.000	33.266	0.029		
Intercept	0.098	1	0.098	13,747.563	0.000	Standard error	0.001
Initial angle	0.000	2	0.000	30.484	0.032		
Overall length	0.000	2	0.000	27.062	0.036	Lower bound	0.100
Cylinder pivot length	0.001	2	0.000	42.250	0.023		
Error	1.422 × 10^−7^	2	7.111 × 10^−6^			Upper bound	0.108
Total	0.099	9					

**Table 6:** Post-inspection of influencing factors.

Group	Parameter	Number of Configurations	Subset	
			1	2
Group 1 (Initial angle)	46.40	3	0.09567	/
	48.33	3	0.10433	0.10433
	50.00	3	/	0.11267
Group 2 (Overall length)	1.45	3	0.09600	/
	1.50	3	0.10467	0.10467
	1.55	3	/	0.11200
Group 3 (Cylinder pivot length)	1.10	3	0.09400	/
	1.04	3	/	0.10467
	1.00	3	/	0.11400

**Table 7:** A comprehensive experiment under an initial angle of 48.33°.

Run Number	Initial Angle/°	Overall Length/m	Cylinder Pivot Length/m
1	48.33	1.50	1.04
2	48.33	1.50	1.00
3	48.33	1.50	1.10
4	48.33	1.45	1.04
5	48.33	1.45	1.00
6	48.33	1.45	1.10
7	48.33	1.55	1.04
8	48.33	1.55	1.00
9	48.33	1.55	1.10

**Table 8:** Swing results of each combination under full factorial experiments.

Run No.	1	2	3	4	5	6	7	8	9
Swing angle (×10^−5^ rad)	7.13	7.56	6.21	6.66	7.24	6.06	7.14	7.84	6.42
Swing amplitude/mm	0.107	0.114	0.093	0.097	0.105	0.088	0.111	0.122	0.100

**Table 9:** Validation test optimization parameter results.

Combination	Initial Angle/°	Overall Length/m	Cylinder Pivot Length/m	Theoretical Amplitude/mm	Actual Amplitude/mm	Optimization Effectiveness	Error
Original parameter	46.40	1.45	1.0	0.0979	0.0990	\	1.11%
Optimized combination 1	48.33	1.45	1.0	0.1050	0.1015	\	3.33%
Optimized combination 2	48.33	1.45	1.1	0.0880	0.0875	11.62%	0.57%

## Data Availability

The data in this article can be made available upon request.
